# Therapeutic Anticoagulation with Argatroban and Heparins Reduces Granulocyte Migration: Possible Impact on ECLS-Therapy?

**DOI:** 10.1155/2020/9783630

**Published:** 2020-04-25

**Authors:** Andre Bredthauer, Manuel Kopfmueller, Michael Gruber, Sophie-Marie Pfaehler, Karla Lehle, Walter Petermichl, Timo Seyfried, Diane Bitzinger, Andreas Redel

**Affiliations:** ^1^Department of Anesthesiology, University Medical Center Regensburg, Regensburg, Germany; ^2^Department of Internal Medicine II, Goldbergklinik Kelheim, Kelheim, Germany; ^3^Department of Cardiothoracic Surgery, University Medical Center Regensburg, Regensburg, Germany; ^4^Department of Anesthesiology, Klinikum St. Marien Amberg, Amberg, Germany

## Abstract

**Introduction:**

Anticoagulants such as argatroban and heparins (low-molecular-weight and unfractionated) play an immense role in preventing thromboembolic complications in clinical practice. Nevertheless, they can also have a negative effect on the immune system. This study is aimed at investigating the influence of these substances on polymorphonuclear neutrophils (PMNs), whose nonspecific defense mechanisms can promote thrombogenesis.

**Methods:**

Blood samples from 30 healthy volunteers were investigated, whereby PMNs were isolated by density gradient centrifugation and incubated with 0.8 *μ*g/mL of argatroban, 1.0 U/mL of low-molecular-weight heparin (LMWH), 1.0 U/mL of unfractionated heparin (UFH), or without drug (control). A collagen-cell mixture was prepared and filled into 3D *μ*-slide chemotaxis chambers (IBIDI® GmbH, Germany). Stimulation was initiated by using a chemokine gradient of n-formyl-methionine-leucyl-phenylalanine (fMLP), and microscopic observation was conducted for 4.5 hours. The cells' *track length* and *track straightness*, as well as the number of attracted granulocytes, level of ROS (reactive oxygen species) production, and NET (neutrophil extracellular traps) formation, were analyzed and categorized into migration distances and time periods.

**Results:**

All three anticoagulants led to significantly reduced PMN track lengths, with UFH having the biggest impact. The number of tracks observed in the UFH group were significantly reduced compared to the control group. Additionally, the UFH group demonstrated a significantly lower track straightness compared to the control. ROS production and NET formation were unaffected.

**Conclusion:**

Our data provide evidence that anticoagulants have an inhibitory effect on the extent of PMN migration and chemotactic migration efficiency, thus indicating their potential immune-modulatory and prothrombotic effects.

## 1. Introduction

Critically ill patients frequently require anticoagulation in order to prevent deep vein thrombosis (DVT) and pulmonary embolism (PE). Patients suffering from organ failure and in need of organ replacement therapy, i.e., receiving treatment with Extra Corporeal Life Support (ECLS) for respiratory or cardiocirculatory distress or hemodialysis for kidney failure, are—even under anticoagulation therapy—at high risk for thrombotic events due to activation of the coagulation system as mediated by the artificial surfaces involved [1–3]. Anticoagulation optimization is, therefore, mandatory in these patients [4, 5]. Clot formation within the membrane lung or the extracorporeal circuit is the most common technical complication, with an incidence of up to 22% [6] during ECLS therapy resulting in high mortality rates despite technical improvements and more sophisticated anticoagulation regimens [7].

In recent years, several studies have provided evidence for the interaction between polymorphonuclear granulocytes (PMNs) and the coagulation system. While forming the first line of innate immunity, imbalanced granulocyte activation can result in autoimmune diseases and thrombotic complications [8–10]. Furthermore, leukocyte deposits can be found in thrombi formed on gas exchange membranes of membrane lungs [11, 12].

Unfractionated heparin (UFH) or low-molecular-weight heparins (LMWH) are usually used for anticoagulation [7]. The selective factor-IIa (thrombin)-inhibitor argatroban is an upcoming alternative which is used for anticoagulation in patients suffering from heparin-induced thrombocytopenia [13]. Although studies have repeatedly shown suppression of granulocyte function in a dose-dependent manner for UFH and LMWH [14–17], far less is known about the impact of argatroban on granulocyte migration [18, 19].

The aim of this study was to investigate the impact and time-dependent viability of UFH, LMWH, and argatroban on the function of isolated granulocytes by performing in vitro assays for the comparative analysis of granulocyte migration capacity, chemotaxis, reactive oxygen species (ROS) production, and neutrophil extracellular trap formation (NETosis).

## 2. Materials and Methods

### 2.1. Granulocyte Preparation

The experimental setup was based on our previous studies [20, 21]. Briefly, whole blood drawn from 30 healthy blood donors (Table 1) with their informed consent (as approved by the local ethics committee, Vote No.: 15-101-0043) was anticoagulated using ethylenediaminetetraacetic acid (EDTA) in a final concentration of 1.6 mg/mL blood. The blood samples were diluted with 4-(2-hydroxyethyl)-1-piperazineethanesulfonic acid (HEPES) and Tyrode solution (modified to 1 mM CaCl_2_ and 1 mM MgCl_2_). PMNs were isolated by density gradient centrifugation (756 g) at an ambient temperature of 21°C for 20 minutes with Lympho Spin Medium on top of Leuko Spin Medium (pluriSelect Life Science, Leipzig, Germany) according to the manufacturer's instructions. After two washing steps, PMNs were resuspended in RPMI 1640 (Pan-Biotech GmbH, Aidenbach, Germany) made up with 10% fetal calf serum (FCS, Sigma-Aldrich GmbH, Steinheim, Germany).

Cells from a single donor were incubated in parallel at 37°C for 30 minutes with 0.8 *μ*g/mL of argatroban (AGATRA, Mitsubishi Tanabe Pharma GmbH, Düsseldorf, Germany), 1.0 IU/mL anti-Xa-activity of the LMWH Enoxaparin (Clexane, Sanofi-Aventis GmbH, Frankfurt, Germany), and 1.0 IU/mL anti-Xa-activity of UFH (HEPARIN ROTEXMEDICA, ROTEXMEDICA GmbH Arzneimittelwerk, Trittau, Germany) or without drug as a control.

### 2.2. Microscopy and Live Cell Imaging

To visualize the production of ROS 1 *μ*M di-hydro-rhodamine (DHR, Invitrogen D632, Sigma-Aldrich GmbH, Steinheim, Germany) and, accordingly, its fluorescence, active oxidized product rhodamine 123 was used. NET formation was detected by staining released extracellular DNA with 0.5 *μ*g/mL of 4′,6-diamidin-2-phenylindol (DAPI, D9542, Sigma-Aldrich GmbH, Steinheim, Germany). Light and fluorescence microscopy was utilized to examine the cells of all four groups (argatroban, LMWH, UFH, and control), which were suspended in type I collagen gel (1.5 mg/mL of PureCol with 1.67% FCS, Advanced BioMatrix Inc., San Diego, CA, USA) and filled into *μ*-Slide Chemotaxis chambers (IBIDI GmbH, Martinsried, Germany) in accordance with the manufacturer's protocol. There is a reservoir on both sides of the chamber's gel channels; one was filled with n-formyl-methionine-leucyl-phenylalanine (fMLP 10 nM; Sigma-Aldrich GmbH, Steinheim, Germany) with RPMI/10% FCS and the other with RPMI with 10% FCS to establish a linear gradient of the chemoattractant. Each channel was filled with cell-gel suspensions preincubated with either argatroban, LMWH, UFH, or none of the aforementioned (control) in order to exclude interdonor variability.

The PMN observation was carried out with a Leica DMi8 (Leica GmbH, Wetzlar, Germany) with an automatically adjustable microscope stage and camera (DFC9000, Leica GmbH, Wetzlar, Germany). To ensure stable test conditions (37°C, 5% CO_2_), a stage top incubator (IBIDI) was used. During the observation period of 4.5 hours, fluorescence and phase contrast images were taken automatically every 30 seconds by the Leica Application Suite X (LAS X 3.0.4.16529, Leica GmbH, Wetzlar, Germany).

### 2.3. Image Data Analysis

Image data analysis was based on previous experiments [20, 21]. Briefly, a total of 540 images per chamber were analyzed using Imaris software (version 9.0.0, Bitplane, Zurich, Switzerland). To evaluate cell migration, the software recognized and tracked migrating cells semiautomatically over a predetermined time period. After monitoring the migration over the entire observation time, the 4.5-hour period was divided into blocks of 120 images per hour and the calculated spots and tracks within these blocks were analyzed. Data was then exported to Excel (Microsoft Corp., Redmond, WA, USA), including the total track length (*μ*m) (length of the migration route of each individual cell) and the track straightness (fraction of Euclid track length and total track length showing the cell's tendency to migrate directly; higher factors refer to straighter lines) of each track. A track straightness above 0.2 and track lengths between 11 *μ*m and 200 *μ*m were required to identify and track granulocytes and exclude artefacts. Furthermore, the number of tracked PMNs was analyzed in categorized migration distances (the blocks of total track lengths were 11-50, 51-100, 101-150, 151-200, 201-250, and above 250 *μ*m).

Relationships were examined through contingency analyses, and crosstabs were created comparing the observed and expected normal distribution values.

Pearson's chi-square test was used to test the significance of the differences between the variables. In terms of the track straightness comparison, a track length above 50 *μ*m was required.

To investigate the time point of maximum ROS production (Tmax), images were taken every 2.5 minutes during the 4.5-hour observation period and the sum of the surface areas with DHR/rhodamine 123 staining was calculated. These surface areas were also recognized semiautomatically by Imaris and exported to Excel files. A third degree polynomial trendline was fitted to the parabolic course (time vs. sum of voxels) to determine the Tmax of ROS (min). Tmax values above 120 min were defined as implausible.

The response of DAPI contact with extracellular DNA (NET formation) was also analyzed by summing up single surface areas per time point, which resulted in sigmoidal curves. The time points at which the half-maximal effects (Et_50_) were reached were calculated by population analysis with Phoenix 64 software (sigmoidal baseline E_max_-model; Certara Inc., Princeton, NJ, USA). ET_50_ values beyond the observational period were defined as implausible.

### 2.4. Flow Cytometry

In addition to the life cell imaging, a portion of the cells was prepared for flow cytometry (FACSCalibur, Becton & Dickinson, Heidelberg, Germany) to analyze the ROS production with a second method and to compare the vitality among the four groups using CellQuest Pro software (version 5.2, Becton-Dickinson Bioscience, San Jose, USA) and FlowJo (version 10.0.7, FlowJo LLC, Ashland, OR, USA). In terms of cell vitality, propidium iodide- (PI-) negative cells were considered vital.

The materials and methods for quantifying ROS production have been described in detail in previous papers [22, 23]. Briefly, cells were preincubated in 500 *μ*L of HEPES buffer and Tyrode solution (modified to 1 mM CaCl_2_ and 1 mM MgCl_2_), 5 *μ*L of DHR (10 *μ*M), and 5 *μ*L of seminaphtharhodafluor (SNARF, 10 *μ*M, Invitrogen). Stimulation was induced by adding either 5 *μ*L of fMLP (10 *μ*M) and 5 *μ*L of human tumor necrosis factor alpha (TNF*α*, 1 *μ*g/mL, PeproTech Inc., Rocky Hill, NJ, USA) or 5 *μ*L of phorbol-12-myristate-13-acetate (PMA, 10 *μ*M, Sigma-Aldrich GmbH). Finally, 5 *μ*L of PI (33671, 1.5 mM, Serva Electrophoresis GmbH, Heidelberg, Germany) was added to detect dead cells.

### 2.5. Statistical Analysis

All Excel data (Microsoft Excel 2016) were subsequently transferred to SPSS Statistics (Version 25, IBM Corp., Armonk, NY, USA), and the Kolmogorov-Smirnov test was used to confirm normal distribution for each group. Where there was a normal distribution, the mean values (MV) were compared, specifying the standard deviation (±SD). Raw data were compared using the Kruskal–Wallis one-way analysis of variance (ANOVA). Where there was variance homogeneity (Levene), post hoc analysis was carried out using the Bonferroni correction or, alternatively, the Dunnett-T3 test. In nonparametric comparison of groups, medians were given with the interquartile range (IQR) and plotted as box plots and lower and upper quartiles, as well as minima and maxima that had been calculated. Contingency analyses, crosstabs, and Pearson's chi-square test were used to detect significant contexts. *p* values below or equal to 0.05 were considered statistically significant.

## 3. Results

**Figure 1:**
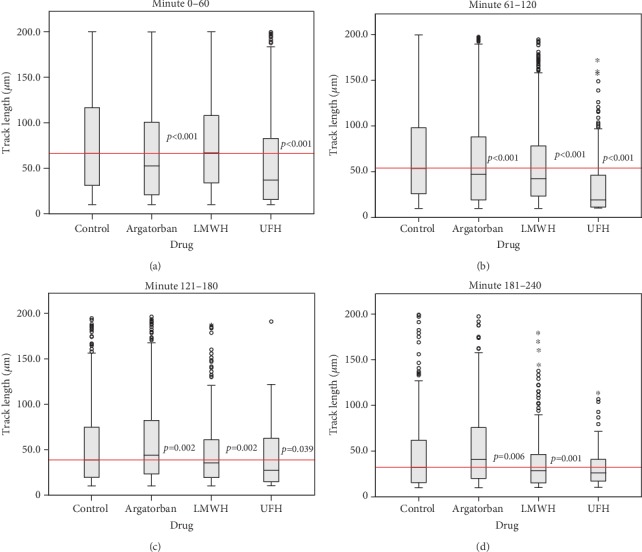
Comparison of track length medians of the verum groups with the control group at the observation time periods: 0–60 min (a), 61–120 min (b), 121–180 min (c), and 181–240 min (d). Cont: control; Arga: argatroban; LMWH: low-molecular-weight heparin; UFH: unfractionated heparin.

The control group (*n* = 2118 tracks) demonstrated a median track length of 66.3 *μ*m (IQR = 85.4 *μ*m) in the first 60 minutes of observation. At 52.6 *μ*m (IQR = 79.7 *μ*m), the median of the argatroban group (*n* = 2101 tracks) was significantly lower than that of the control group (*p* < 0.001). The LMWH group (*n* = 1922 tracks) had a median of 66.9 *μ*m (IQR = 74.0 *μ*m), thus not differing from the control, while the lowest median of 37.0 *μ*m (IQR = 67.3 *μ*m) (*p* < 0.001) was found in the UFH group (*n* = 524 tracks) (Figure 1(a)). Nevertheless, it was possible to observe significant differences between the argatroban and LMWH groups versus the UFH group (*p* < 0.001).

Between minutes 61 and 120, all three verum groups differed significantly to the control (*n* = 1198 tracks), which had a median of 53.6 *μ*m (IQR = 72.0 *μ*m) (*p* < 0.001). For the argatroban group (*n* = 989 tracks) and the LMWH group (*n* = 897 tracks), the median was 47.3 *μ*m (IQR = 68.7 *μ*m) and 42.6 *μ*m (IQR = 55.0 *μ*m), respectively. As before, the lowest value was found in the UFH group (*n* = 204 tracks) with a median of 19.3 *μ*m (IQR = 34.7 *μ*m), which also differed from the LMWH and the argatroban groups (*p* < 0.001) (Figure 1(b)).

From minute 121 to 180, there were no differences between the control (*n* = 687 tracks), which had a median of 38.8 *μ*m (IQR = 55.2 *μ*m), and the argatroban group (*n* = 627 tracks), with a median of 43.8 *μ*m (IQR = 59.3 *μ*m), or the LMWH group (*n* = 386 tracks) with a median of 35.4 *μ*m (IQR = 42.0 *μ*m). Only the UFH group (*n* = 79 tracks) differed from the control with a significantly lower median of 27.3 *μ*m (IQR = 48.0 *μ*m) (*p* = 0.039) (Figure 1(c)). The median of the argatroban group was significantly higher than the medians of the LMWH and the UFH groups (*p* = 0.002).

Between minutes 181 and 240, the argatroban group (*n* = 412 tracks) was—with a median of 40.8 *μ*m (IQR = 55.8 *μ*m)—the only group that demonstrated a significantly higher median than the control. The control (*n* = 285 tracks) had a median of 32.0 *μ*m (IQR = 46.6 *μ*m) (*p* = 0.006) and the LMWH group (*n* = 146 tracks) had a median of 28.6 *μ*m (IQR = 31.5 *μ*m) (*p* = 0.001). There were no differences between the other groups (the UFH group (*n* = 35 tracks) showed a median of 26.1 *μ*m (IQR = 24.8 *μ*m)) (Figure 1(d)). No further significant differences could be detected in the last 30 minutes of the observation period (data not shown).

The crosstab containing the observed track counts in defined length categories, and the expected number of tracks which were calculated based on an assumed normal distribution, revealed a significant correlation between drug and number of tracks (chi-square (15) = 165, *p* < 0.001, *n* = 13552 tracks). The comparison showed no significant deviations in the control and the argatroban groups, for which the relative deviations were in the range of -1.82 and -4.32%. For all distance categories above 151 *μ*m (-7.77 and -22.1%), deviations tended to be of higher magnitude in the LMWH group but did not reach significance. The highest discrepancies between the observed and expected counts were found in the UFH group. Here, deviations ranged from -23.0 to -72.9% in the categories beyond 51 *μ*m total track length, indicating that the relative number of nonmigrating granulocytes (total TL < 50 *μ*m; mean + 35.4%) is elevated and the relative number of active granulocytes (total TL > 51 *μ*m; mean -44.3%) is reduced (Table 2).

**Figure 2:**
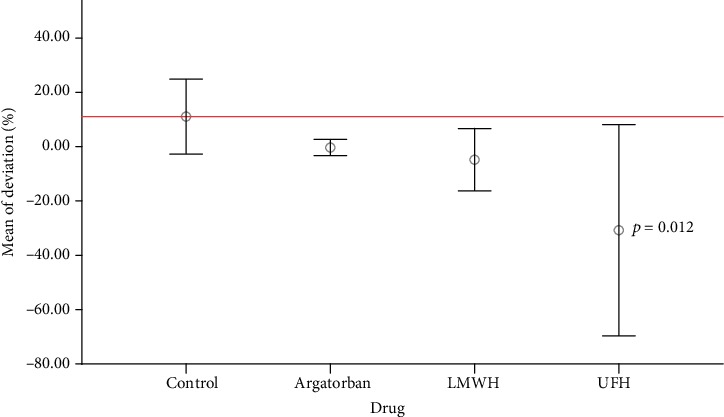
Number of tracked granulocytes: there were no significant differences in the relative deviations between the argatroban, LMWH, and control groups. Only the UFH group differed significantly from the control (*p* = 0.012). LMWH: low-molecular-weight heparin; UFH: unfractionated heparin.

Consequently, only the mean granulocyte counts of the UFH group (*n* = 869 tracks) showed a significant deviation of -31.1 ± 36.9% between the tracked and expected cells per track length categories, compared to the control group (*n* = 4657 tracks) with only 10.6 ± 13.1% deviation (*p* = 0.012) (Figure 2).

The comparison of the track straightness showed no differences between the control (*n* = 3139 tracks), which had a median track straightness of 0.40 (IQR = 0.34), and the argatroban group (*n* = 2778 tracks) with a median of 0.38 (IQR = 0.32). This also applied to the LMWH group (*n* = 2307 tracks) with a median of 0.40 (IQR = 0.33). The straightness of the UFH group (*n* = 430 tracks), however, differed significantly from the control (*p* < 0.001) with a median of 0.32 (IQR = 0.32).

**Figure 3:**
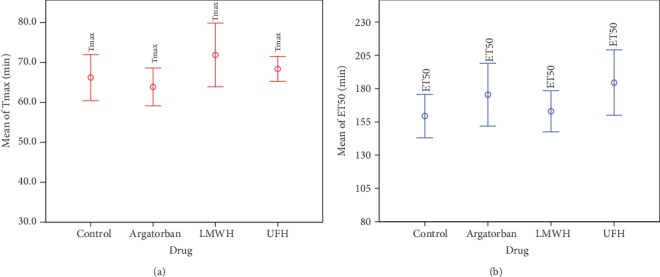
Neither the response of oxidized DHR nor the time course of DAPI-free DNA complex formation showed statistically significant mean differences between the four groups. NET-forming (ET_50_, blue) and ROS production (Tmax, red). LMWH: low-molecular-weight heparin; UFH: unfractionated heparin.

After excluding one implausibly extreme value, the fluorescence response of oxidized DHR in life cell imaging did not differ from the mean Tmax of the control (66.2 ± 15.5 min, *n* = 30). Each group demonstrated the following Tmax values: the argatroban group (63.9 ± 12.7 min, *n* = 30), the LMWH group (71.8 ± 14.4 min, *n* = 15), and the UFH group (68.3 ± 5.45 min, *n* = 14) (Figure 3).

**Figure 4:**
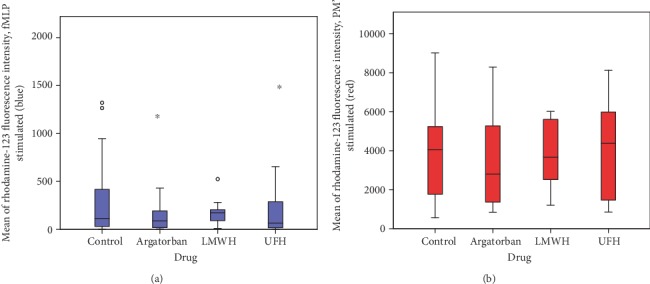
FACS analysis of ROS production: comparisons between fMLP (blue) and PMA- (red-) stimulated explorations of the four groups showed no differences. LMWH: low-molecular-weight heparin; UFH: unfractionated heparin.

After excluding five implausible measurements with ET_50_values above 400 min, more than eleven ET_50_ values could be evaluated for each group. The mean ET_50_ values were as follows: the control (159 ± 37.8 min, *n* = 23), the argatroban group (175 ± 53.2 min, *n* = 22), the LMWH group (163 ± 25.5 min, *n* = 13), and the UFH group (185 ± 36.6 min, *n* = 11) (Figure 4).

In the FACS analysis of the fMLP-stimulated granulocytes, the control (*n* = 22) showed a median rhodamine 123 (ROS production) fluorescence intensity of 115 (IQR = 437), the argatroban group (*n* = 22) a median of 89 (IQR = 193.5), the LMWH group (*n* = 10) a median of 175 (IQR = 139), and the UFH group (*n* = 12) a median of 66 (IQR = 362). In the positive control (PMA-stimulated), a median of 4254 (IQR = 3456) was obtained in the control group (*n* = 22), and medians of 3390 (IQR = 3847), 3889 (IQR = 3125), and 4555 (IQR = 4472) were obtained in the argatroban group (*n* = 22), the LMWH group (*n* = 10), and the UFH group (*n* = 12), respectively. Thus, all medians of fluorescence intensity caused by ROS production were found to be indistinguishable (Figure 4).

After the 30-minute preincubation period with the drugs, cells of the argatroban group (*n* = 23) showed a median of 98.6% (IQR = 1.50%) vital granulocytes. In the LMWH group (*n* = 11) with a median of 98.7% (IQR = 2.15%), vital cells could be found, which was similar to that of the UFH group (*n* = 12) with a median of 98.8% (IQR = 2.30%). Therefore, compared to the control (*n* = 23) with a median of 98.8% (IQR = 2.00), no significant differences could be observed.

## 4. Discussion

This study investigated the impact of the clinically used anticoagulants UFH, the LMWH Enoxaparin, and the direct thrombin-inhibitor argatroban on isolated granulocytes in terms of granulocyte migration, time dependency of ROS production and NET formation, and vitality.

PMNs were obtained from healthy volunteers of both sexes without prior mobilization and were then incubated in parallel with UFH, LMWH, and argatroban after two washing steps in order to exclude any external influences on baseline granulocyte function, e.g., infections or humoral and hormonal factors. Therefore, measurements of humoral factors such as clotting factors, chemokines, or cytokines were not performed. All three anticoagulants are known to reduce inflammatory mediators produced by blood cells and other tissues (e.g., IL-6, IL-8, TNF*α*, or nuclear factor kappa B (NF*κ*-B)) after longer incubation times, ranging from three hours to four weeks [18, 24, 25].

UFH is used in clinical routine since 1971. It activates antithrombin and exerts its anticoagulatory effects by facilitating the interaction between antithrombin (AT) and thrombin, as well as the interaction with coagulation factor X [26]. LMWH directly inhibits coagulation factor X, creating a dose-dependent anticoagulant effect. It has also been used in clinical routine for two decades, and its use has been proven safe in medical and surgical patients [27, 28]. Argatroban is a direct thrombin inhibitor used to manage anticoagulation during HIT II [13]; its feasibility for anticoagulation during ECLS and hemodialysis has already been proven [29, 30].

The dosages of the three anticoagulants under investigation were chosen to match clinical routine. According to the manufacturer, an activated prothrombin time (aPTT, baseline 27-36 seconds) prolonged by a factor of 1.5 to 3 is required for therapeutic anticoagulation with argatroban [31]. Plasma levels of 0.8 *μ*g/mL of argatroban result in an aPTT of 62-65 seconds [32]. The dosage for the LMWH Enoxaparin was chosen according to the manufacturer's prescription information for therapeutic anticoagulation. After a subcutaneous application of 1 mg/kg bodyweight twice daily, plasma levels of anti-Xa-activity of 1.1 IU/mL can be measured in the steady state [33]. An aPTT prolonged by a factor of 1.5 to 2.5 is required for therapeutic anticoagulation with UFH [34]. The concentration of UFH was chosen based on the same parameters as for Enoxaparin, with—due to ethical considerations—an added 1 IU/mL of anti-Xa-activity to avoid having to administer an UFH infusion to healthy volunteers for several hours in order to achieve stable aPTT values. Furthermore, adjustment of UFH dosage based on anti-Xa assays has been proven more reliable than aPTT-based assays in terms of achieving a faster rate of therapeutic anticoagulation with less variance [35].

For the migration assay, neutrophil activation was initiated by using the chemoattractant fMLP. Stimulation with fMLP has been proven in several studies to be a practicable way to induce NET formation [36]. It enabled the observation of the cellular events in our live cell imaging setting, which was a prerequisite for the precise evaluation of migration. We decided against activation with PMA for the live cell imaging because of the agent's already known substantial impact on granulocyte function [37]. In our flow cytometric assay, granulocyte activation for ROS production was achieved using fMLP, with the addition of PMA to serve as a positive control [38]. Unpublished data from our laboratory reveal an approximate 50% ET_50_ reduction with a PMA concentration above 10 nM.

Migration assays were performed using a collagen I matrix to mimic the physiological extracellular environment. Our collagen I-based experimental set-up enabled live cell imaging and parallel testing of migration, ROS production, and NETosis [20, 21]. In addition, a well-established method of FACS analysis was used for respiratory burst measurement [22, 23].

All three anticoagulants under investigation significantly reduced neutrophil migration compared to the control group. While argatroban already began to suppress PMN migration within the first observation period, LMWH took longer to exert its effect. The biggest impact was measured in the UFH group during the first 180 minutes of the experiment. Furthermore, UFH preincubated granulocytes showed significantly lower relative track numbers of migrating cells compared to the control. Additionally, track straightness was reduced significantly, resulting in less efficient migration in the UFH group. Heparin and LMWH are known to reduce granulocyte migration in various clinical settings. In other studies, researchers found reduced granulocyte infiltration after traumatic brain injury when using the same dosing regimen for UFH and LMWH [39, 40]. Our observations match the findings of these studies, despite our study having used a different matrix. These inhibitory effects have been attributed to stimuli unrelated to the anticoagulant capacities of UFH and LMWH, such as inhibition of thrombin, Mac-1, interaction with heparin-binding proteins, NF*κ*-B, growth factors, and cytokines [39, 40]. After the administration of argatroban, a lower infiltration of granulocytes into the brain tissue of rats suffering traumatic brain injury was observed; another study showed reduced granulocyte infiltration into the liver tissue of rats suffering from fatty liver disease [18, 19]. Both studies postulate that this effect is due to thrombin inhibition, which leads to increased plasma levels in both the brain and liver tissue and serves as a potent activator of granulocytes. After the administration of argatroban in rats with sepsis, Fuchs et al. observed improved intestinal microcirculation with reduced leucocyte adhesion and improved capillary perfusion [41]. These effects were also attributed to thrombin inhibition and subsequent reduction of microthrombi, as well as reduced endothelial activation with less leukocyte adherence. Thrombin also has direct proinflammatory effects due to its receptor (protease-activated receptor (PAR)), resulting in increased levels of AKT, NF*κ*-B, and caspase-3 [42]. In a recent study by Bulani et al., a significant improvement in diabetic cardiomyopathy could be detected [43]. The administration of argatroban reduced granulocyte migration in the first 120 minutes of both experiments. Surprisingly, in the present work, neutrophils incubated with argatroban showed greater migration distances after 120 minutes compared to the control, though migration during this time period was significantly reduced compared to baseline values for both groups.

In our study, no significant differences in ROS production between the groups investigated could be observed, whether it concerned fluorescence intensity or its chronological sequence. This result is in line with the findings of a study by Xu et al.; the researchers could not observe reduced ROS production following a daily subcutaneous administration of 1.5 mg of Enoxaparin in a rat model of liver fibrosis and splenectomy [44]. Xu et al. speculated that Enoxaparin had an inhibitory effect on thrombin and platelets. In contrast, Li and coworkers showed reduced ROS production in cell cultures of the bronchial epithelial cell line HBE 16 after incubation with 50 to 450 IU/mL of UFH [45]. Li et al.'s study postulated that the inhibitory effects of UFH on intracellular signaling pathways are unrelated to the anticoagulant effects responsible for this observation. Hence, the dosages used to achieve reduced ROS production are far beyond the plasma levels of UFH used in our study and far beyond clinical routine.

Concerning the applied dosages of the three substances investigated in our experiment, no significant NET formation differences between the groups could be found. Contrary findings regarding the absolute amounts, but not the chronological sequence, were made by other research groups in studies on UFH and LMWH. Fuchs et al. observed significantly reduced levels of NETs after administration of high-dose UFH at 100 *μ*g/mL, which is equivalent to approximately 10 IU/mL [15]. Reduced NET formation was observed by Manfredi et al. for an identical dose of 1 IU/mL of Enoxaparin [17]. This contrary effect observed in the latter study may be due to different handling regimens concerning how the PMNs were obtained and isolated. In Manfredi et al.'s study, granulocytes were incubated for 30 minutes at 4°C with Enoxaparin, which may have had an inhibitory effect due to hypothermia. To the best of our knowledge, no literature on the interaction of argatroban and NET formation is currently available.

Finally, in our experimental setting, none of the three substances under investigation affected the study's viability. Although UFH is known to be able to induce apoptosis in granulocytes in a dose-dependent manner via the CD61b pathway, plasma levels above 20–50 IU/mL are required to have a significant effect [14, 16, 45]. As previously mentioned, this dosage is far beyond a rational anticoagulation regime.

Further studies are needed to elucidate the different or additional effects due to PMNs coming into contact with artificial surfaces or the shear force effects induced by extracorporeal systems.

## 5. Conclusion

In conclusion, our in vitro study with a collagen-I-matrix provides evidence that the PMN activity sequence is initialized with migration before the cells start to change their shape and produce ROS followed by NET formation. Our data show that anticoagulants in therapeutic dosage, especially UFH, have an inhibitory effect on the ability of granulocytes to migrate, as well as the extent of migration and the ability to migrate efficiently. Anticoagulants might, therefore, have immune modulatory effects. It could be hypothesized that PMNs are less able to migrate in anticoagulated patients. Furthermore, it could be speculated that PMNs of anticoagulated patients could stick to artificial surfaces due to their reduced migration capacity, resulting in a prothrombotic state that might affect therapy with extracorporeal devices. Other granulocyte functions were not affected, and the available literature suggests that higher doses of anticoagulants are required for the inhibition of these functions.

## Figures and Tables

**Table 1:** Characteristics of the blood donors.

Characteristic	Value^∗^
Number of experiments	30
Sex of subjects	16 females, 14 males
Age (years)	24 (19-58)
Height (cm)	170 (155-193)
Weight (kg)	70 (53-108)
BMI	24.9 (19-29)

^∗^Data are expressed as the median (range).

**Table 2:** Comparison of numbers between tracked and expected granulocytes per track length category: number of tracks of the UFH group shows the greatest deviations compared to the control group.

Number of tracked granulocytes in categorized migration distances (track length)										
Total track length (*μ*m)			11-50	51-100	101-150	151-200	201-250	>250	Total	Mean
Observation groups	Control	Number of tracks	2042	1220	722	371	174	128	4657	776.2
		Expected number	2208.2	1218.5	663.2	317.9	150.2	99.0	4657	776.2
		Difference (%)	-7.53	0.12	8.86	16.7	15.9	29.3	63.4	10.6
	Argatroban	Number of tracks	2184	1131	608	299	144	96	4462	743.7
		Expected number	2115.8	1167.5	635.5	304.6	143.9	94.8	4462.1	743.7
		Difference (%)	3.23	-3.13	-4.32	-1.82	0.08	1.24	-4.72	-0.79
	Enoxaparin	Number of tracks	1642	1020	519	218	106	59	3564	594
		Expected number	1690.0	932.6	507.6	243.3	114.9	75.7	3564.1	594
		Difference (%)	-2.84	9.38	2.25	-10.4	-7.77	-22.1	-31.5	-5.25
	UFH	Number of tracks	558	175	81	37	13	5	869	144.8
		Expected number	412.1	227.4	123.8	59.3	28.0	18.5	869.1	144.8
		Difference (%)	35.4	-23.0	-34.5	-37.6	-53.6	-72.9	-186.2	-31.0

## Data Availability

The datasets generated during and/or analyzed during the current study are available from the corresponding author on reasonable request.
